# Characterization of a Soluble Phosphatidic Acid Phosphatase in Bitter Melon (*Momordica charantia*)

**DOI:** 10.1371/journal.pone.0106403

**Published:** 2014-09-09

**Authors:** Heping Cao, Kandan Sethumadhavan, Casey C. Grimm, Abul H. J. Ullah

**Affiliations:** U.S. Department of Agriculture, Agricultural Research Service, Southern Regional Research Center, New Orleans, Louisiana, United States of America; University of Kansas School of Medicine, United States of America

## Abstract

*Momordica charantia* is often called bitter melon, bitter gourd or bitter squash because its fruit has a bitter taste. The fruit has been widely used as vegetable and herbal medicine. Alpha-eleostearic acid is the major fatty acid in the seeds, but little is known about its biosynthesis. As an initial step towards understanding the biochemical mechanism of fatty acid accumulation in bitter melon seeds, this study focused on a soluble phosphatidic acid phosphatase (PAP, 3-sn-phosphatidate phosphohydrolase, EC 3.1.3.4) that hydrolyzes the phosphomonoester bond in phosphatidate yielding diacylglycerol and P_i_. PAPs are typically categorized into two subfamilies: Mg^2+^-dependent soluble PAP and Mg^2+^-independent membrane-associated PAP. We report here the partial purification and characterization of an Mg^2+^-independent PAP activity from developing cotyledons of bitter melon. PAP protein was partially purified by successive centrifugation and UNOsphere Q and S columns from the soluble extract. PAP activity was optimized at pH 6.5 and 53–60°C and unaffected by up to 0.3 mM MgCl_2_. The K*_m_* and V*_max_* values for dioleoyl-phosphatidic acid were 595.4 µM and 104.9 ηkat/mg of protein, respectively. PAP activity was inhibited by NaF, Na_3_VO_4_, Triton X-100, FeSO_4_ and CuSO_4_, but stimulated by MnSO_4_, ZnSO_4_ and Co(NO_3_)_2_. In-gel activity assay and mass spectrometry showed that PAP activity was copurified with a number of other proteins. This study suggests that PAP protein is probably associated with other proteins in bitter melon seeds and that a new class of PAP exists as a soluble and Mg^2+^-independent enzyme in plants.

## Introduction

*Momordica charantia* is often called bitter melon, bitter gourd or bitter squash because its fruit has a bitter taste. It is a tropical and subtropical vine of the *Cucurbitaceae* family and widely grown in Asia, Africa and the Caribbean. The plant grows as herbaceous, tendril-bearing vine up to 5 m long. Bitter melon flowering occurs during June-July and fruit develops during September-November in the Northern Hemisphere. The fruit has a distinct warty exterior and an oblong shape. It is hollow in cross-section with a relatively thin layer of flesh surrounding a central seed cavity filled with large, flat seeds and pith. The fruit is generally consumed in the green or early yellowing stage. The fruit’s flesh is crunchy and watery in texture and tasted bitter at these stages. The skin is tender and edible. Seeds and pith appear white in unripe fruits, are not intensely bitter and can be removed before cooking. Bitter melon is often used in Chinese cooking for its bitter flavor, typically in stir-fries, soups and herbal teas. It has also been used as the bitter ingredient in some Chinese and Okinawan beers. Bitter melon seeds are rich in fatty acids and minerals including iron, beta carotene, calcium, potassium and many vitamins. The fatty acid compositions of bitter melon oil include 37% of saturated fatty acids mainly stearic acid; 3% of monounsaturated fatty acid dominantly linoleic acid, and 60% of polyunsaturated fatty acid predominately alpha-eleostearic acid (α-ESA, 9*cis*, 11*trans*, 13*trans* octadecatrienoic acid) which counts for 54% of the total fatty acids [1].

Bitter melon has been used as herbal medicine in Asia and Africa for a long time. It has been used as an appetite stimulant, a treatment for gastrointestinal infection, and to lower blood sugar in diabetics in traditional Chinese medicine. Recent studies have demonstrated the potential uses of bitter melon oil with a wide range of nutritional and medicinal applications because of its anti-cancer effect [2]–[10], anti-diabetic activity [11]–[19], anti-inflammatory effect [20], antioxidant activity [21]–[23], anti-ulcerogenic effect [24]–[26] and wound healing effect [27]. Alpha-ESA, a conjugated linolenic acid, may be the key bioactive compound in the seed oil. Alpha-ESA from bitter melon seeds has cytotoxic effect on tumor cells [6], induces apoptosis and upregulates GADD45, p53 and PPARγ in human colon cancer Caco-2 cells [3], blocks breast cancer cell proliferation and induces apoptosis through a mechanism that may be oxidation dependent [2], protects plasma, low density lipoprotein and erythrocyte membrane from oxidation which may be effective in reducing the risk of coronary heart disease in diabetes mellitus [28] and unregulates mRNA expression of PPARα, PPARγ and their target genes in C57BL/6J mice [29]. These studies suggest that α-ESA has anti-cancer, anti-diabetic, and anti-inflammatory activities, inhibits tumor cell proliferation, lowers blood fat and prevents cardiovascular diseases.

**Figure 1:**
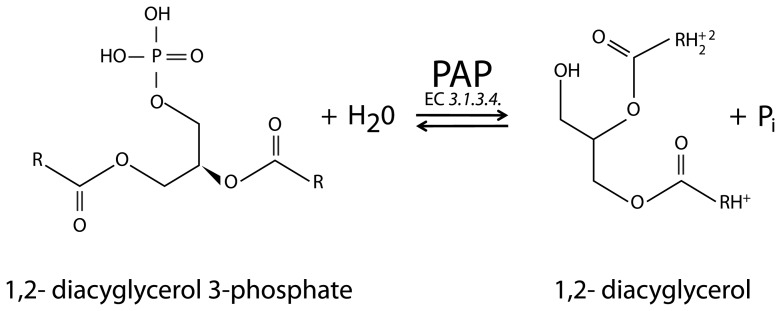
The schematic representation of enzymatic reaction catalyzed by PAP. The enzyme hydrolyzes the phosphoester linkage of PtdOH and generates DAG and P_i_.

Currently, little is known about the enzymatic mechanism for the biosynthesis of α-ESA in bitter melon seeds. In general, acyltransferases including diacylglycerol transferases [30], [31], add fatty acyl groups sequentially to the sn-1, sn-2 and sn-3 positions of glycerol-3-phosphate (G3P) to form triacylglycerol (TAG). This pathway is commonly referred to the Kennedy or G3P pathway [32]. A key step in TAG biosynthesis is the dephosphorylation of the sn-3 position of phosphatidate (PtdOH) catalyzed by phosphatidic acid phosphatase (PAP or lipins) to produce diacylglycerol (DAG) and inorganic phosphate (P_i_) (Figure 1) [33]. PtdOH is synthesized by the actions of glycerophosphate acyltransferase (GPAT) and lysophosphatidic acid acyltransferase (LPAAT). DAG formation is believed to be the penultimate key step in Kennedy pathway because it is a critical metabolite for the synthesis of TAG, phosphatidylethanolamine (PtdEtn), and phosphatidylcholine (PtdCho) [33]–[35].

As an initial step towards understanding the biochemical mechanism of fatty acid accumulation in bitter melon seeds, we focused our studies on PAP (3-sn-phosphatidate phosphohydrolase, EC 3.1.3.4) that dephosphorylates phosphatidic acid (PA, also called PtdOH) to generate DAG and P_i_. PAP family enzymes are currently classified as either soluble PAP [36] or membrane-bound PAP [37]. Based on the requirement of Mg^2+^ for activity, the enzyme could also be divided into 2 classes: Mg^2+^-dependent and Mg^2+^-independent PAP [38]. Typically, soluble PAP is Mg^2+^-dependent; whereas membrane-bound PAP is Mg^2+^-independent. We recently identified a soluble PAP in bitter melon cotyledons [39]. We report here the partial purification and characterization of PAP from developing cotyledons of bitter melon as a soluble and Mg^2+^-independent enzyme.

## Results and Discussion

### Subcellular distribution of PAP activity in bitter melon cotyledons

**Figure 2:**
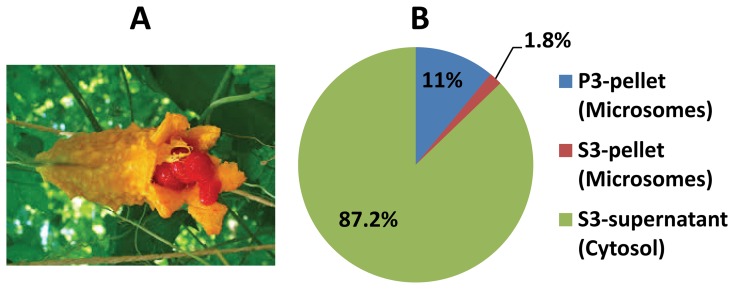
Subcellualr distribution of PAP activity in bitter melon cotyledons. (A) bitter melon of Indian origin was used in the study. (B) Distribution of PAP activity in bitter mellow cotyledons. The cotyledon extract was successively centrifuged at 3,000 *g*, 18,000 *g* and 105,000 *g*. The final pellet (P3-pellet) after ultracentrifugation is generally regarded as the microsomal membranes. Following dialysis and centrifugation of the 105,000 *g* supernatant, the S3-pellet (contaminated microsomes) and the S3-supernatant (cytosol) were obtained. All three fractions were analyzed for PAP activity. The data presented are the mean of 2 assays for each sample.

PAP family enzymes are currently classified as either soluble PAP or membrane-bound PAP [36]. Differential centrifugation was used to separate the cytosol and microsomal membrane fractions for determining the subcellular localization of PAP activity in *Momordica charantia* (bitter melon, bitter gourd or bitter squash). The cotyledon extract was successively centrifuged at 3,000 *g*, 18,000 *g* and 105,000 *g*. The final pellet and supernatant after ultracentrifugation are generally regarded as the microsomal membranes and the cytosol, respectively [40]. PAP activity in the 105,000 *g* pellet (P3-pellet) was only 11% of the total PAP activity (Figure 2). Following dialysis and centrifugation of the supernatant, the S3-pellet contained only 1.8% whereas the S3-supernatant contained 87.2% of the total activity (Figure 2). These subcellular distributions of PAP activity clearly demonstrated that the great majority of PAP activity in bitter melon seeds was soluble and localized in the cytosol.

### Purification of PAP

PAP protein was partially purified from bitter melon cotyledons by a combination of Q and S columns. Enzyme activity was determined by two assays: PAP and phosphatase activity assays. By PAP activity assay, separation of the soluble PAP with Q column resulted in 3.2-fold of purification with a recovery of 90.3% of total PAP activity (Table 1). The PAP protein did not bind to S column effectively and 83.3% of PAP activity was recovered in the flow-through. Separation of the proteins from the flow-through by a second Q column slightly increased the purity with 1.2-fold of specific activity compared to the load and ¾ of the total activity was recovered (Table 1). After changing the binding condition, some PAP protein was bound to S column which resulted in better purification (3-fold) but this step reduced the yield of PAP in the elution to 20% of the load (Table 1). This scheme of purification generated PAP proteins with an overall purification factor of 12.5-fold and a yield of 11.4% using PAP activity assay (Table 1). A higher purification factor (16-fold) and higher yield (14.5%) of PAP purification was obtained from the same protein samples when using phosphatase activity assay (Table 1). Further purification with Affigel Blue resin did not result in any improvement of purity and actually reduced the total activity of PAP (data not shown). The yield and specific activity of PAP from bitter melon is much higher than those of PAP purified from *Lagenaria siceraria* (bottle gourd, opo squash or long melon) [41]. Additional purification steps resulted in significant loss of activity and the protein yield was low, probably due to PAP-associated with other proteins (see below).

**Table 1:** Purification of PAP from bitter melon cotyledons.

Purification step	Protein (mg)	Volume (mL)	PAP activity				Phosphtase activity			
			Total activity (ηKat)	Specific activity (ηKat/mg/min)	Purification factor	Yield (%)	Total activity (ηKat)	Specific activity (ηKat/mg/min)	Purification factor	Yield (%)
1. Supernatant	276	67	411	1.5	1	100	2928	10.6	1	100
2. 1^st^ Q column flow-through	192	107	13	0.1	0.07	3.2	1220	6.4	0.6	41.7
3. 1^st^ Q column elute	79	33	371	4.7	3.2	90.3	1716	21.7	2.0	58.6
4. 1^st^ S column flow-through	57	47.5	309	5.4	3.6	75.2	1183	20.8	2.0	40.4
5. 2^nd^ Q column elute	35	42	235	6.7	4.5	57.2	936	26.7	2.5	31.96
6. 2^nd^ S column elute	2.5	1.5	47	18.7	12.5	11.4	425	170.0	16.0	14.5

### Linearity of PAP assays

**Figure 3:**
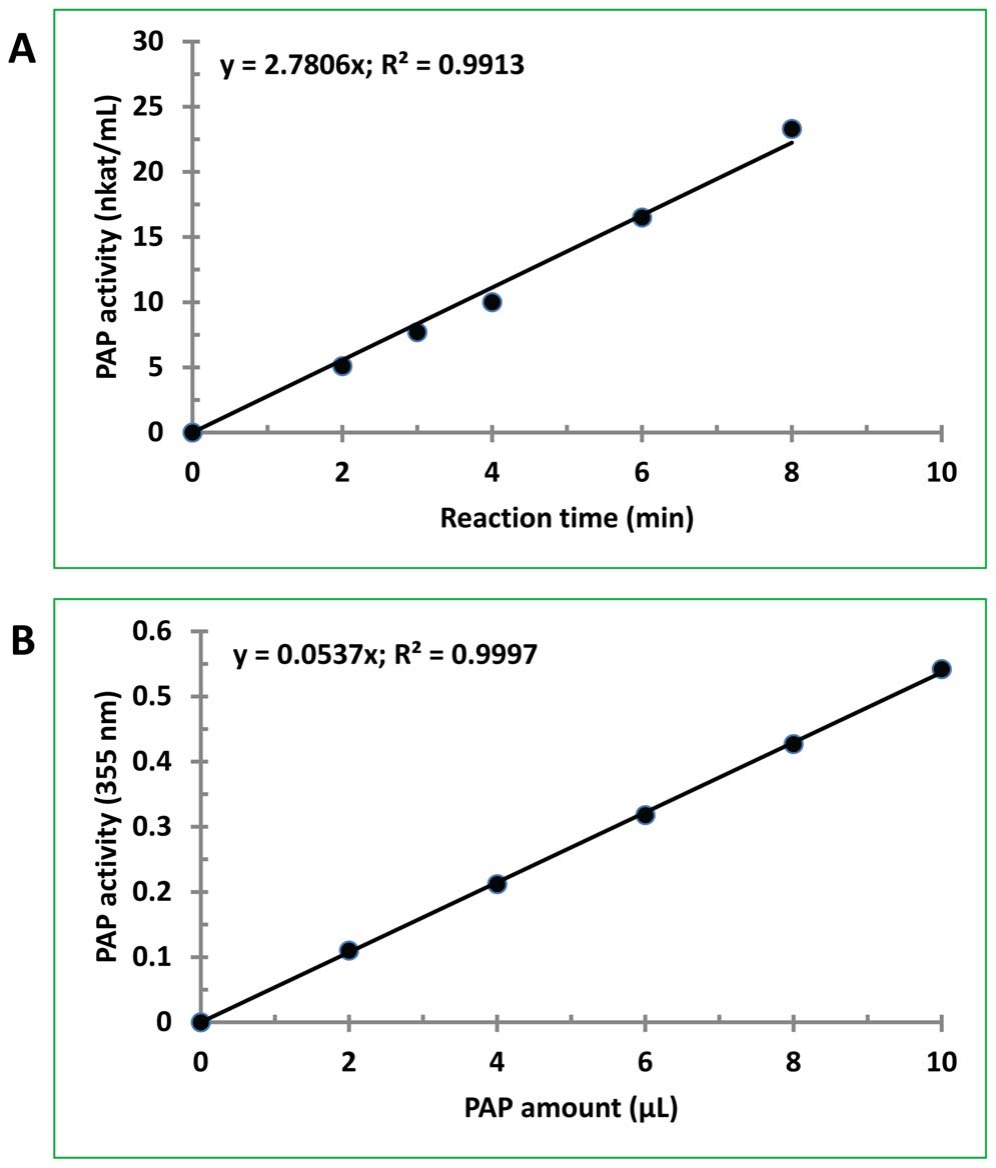
Linearity of PAP assays. PAP activity was assayed using aliquots of the proteins from the second S column fraction duing PAP purification. (A) PAP activity vs. reaction time. The assay was performed at 53°C for various times with 500 µM DPA and 0.3 mM MgCl_2_. Aliquots of the enzymatic reactions were withdrawn for measurement at the indicated time points. (B) PAP activity vs. amount of enzyme. The assay was performed at 53°C using various amounts of the PAP preparation with 500 µM DPA and 0.3 mM MgCl_2_. The data presented are the mean of 2 assays for each sample.

In the initial characterization of the partially purified PAP enzyme, linearity between the incubation time and PAP activity was observed in 50 mM imidazole buffer containing 0.3 mM MgCl_2_, pH 6.5 (Figure 3A). Similar linearity of enzymatic reaction was observed in responding to the amount of enzymes used in the assay (Figure 3B). Data fitting showed that the coefficient of determination (R2) was over 0.99 in both analyses, suggesting a great correlation between PAP activity and reaction time or amount of enzyme used. These assay results suggest that the established assays are suitable for characterization of the partially purified PAP from bitter melon.

### The pH and temperature optima of PAP

**Figure 4:**
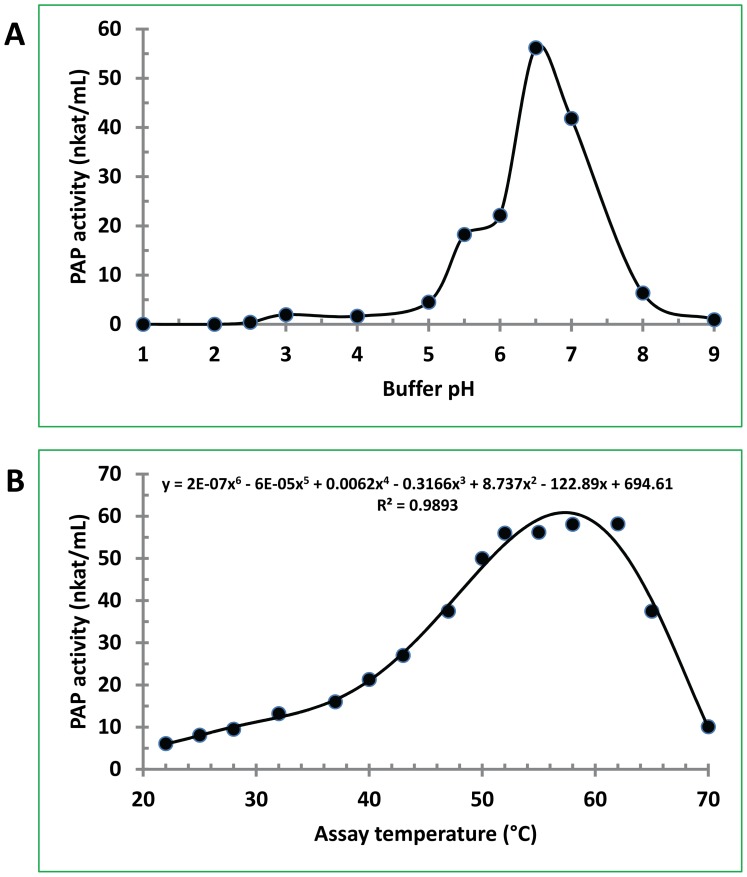
Effect of buffer pH and assay temperature on PAP activity. The assay was performed at 53°C for 30 min using 5 µL of the PAP preparation with 500 µM DPA and 0.3 mM MgCl_2_. (A) pH profile of PAP enzyme catalyzing PtdOH. (B) Temperature profile of PAP enzyme catalyzing PtdOH. The data presented are the mean of 2 assays for each sample.

The pH optima of PAP was observed at pH 6.5 under the assay conditions using 5 µL of the enzyme and 500 µM dioleoyl-phosphatidic acid (DPA) in 50 mM imidazole buffer containing 0.3 mM MgCl_2_ (Figure 4A). The temperature optima were ranged from 53 to 60°C (Figure 4B). These optimal pH and temperature values are slightly higher than those obtained from the crude extract [39]. It is not clear from this study why the temperature optimum is unusually higher than other metabolic enzymes such as the soluble starch synthases from maize kernels [42].

### Mg^2+^-independent activity of PAP

**Figure 5:**
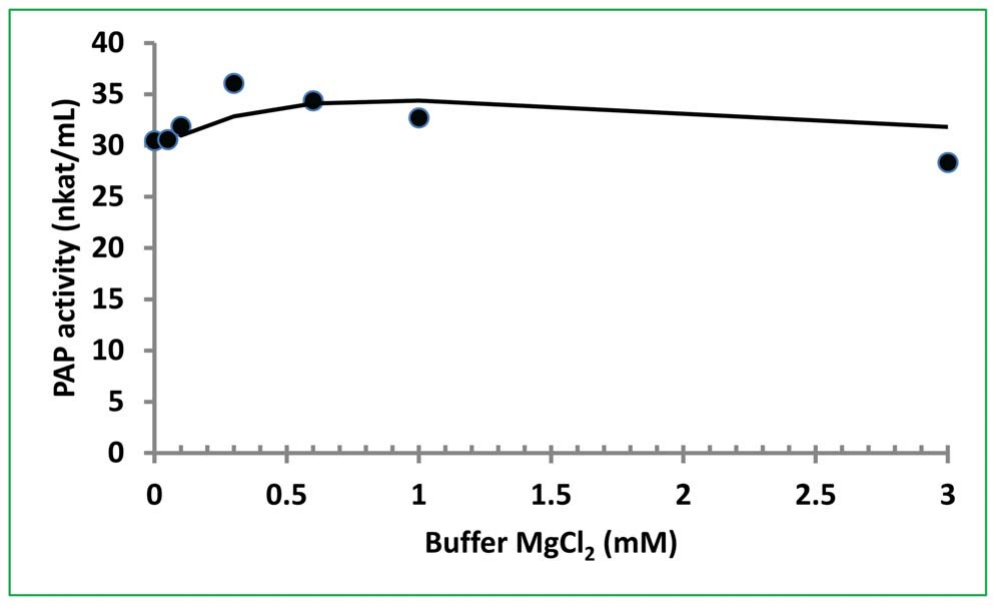
Effect of Mg^2+^ on PAP activity catalyzing PtdOH. The assay was performed at 53°C, pH 6.5 for 30 min using 5 µL of the PAP preparation with 500 µM DPA and various concentrations of MgCl_2_. The data presented are the mean of 2 assays for each sample.

Based on the requirement of Mg^2+^ for activity, PAP family enzymes including lipins and lipid phosphate phosphatases (LPPs) are divided into 2 classes: Mg^2+^-dependent PAP and Mg^2+^-independent PAP [38]. It is known that yeast and invertebrates have a single lipin ortholog, but plants have two PAP/lipin genes and mammals have three lipin genes [33]. The lipin family members of PAP are soluble enzymes and require Mg^2+^ for their activity. LPPs also exhibit PAP activity but they are structurally unrelated to lipin proteins. LPPs are localized to the plasma membrane and do not require Mg^2+^ for their activity. We determined the effect of Mg^2+^ on PAP activity using the purified PAP. Our results showed that PAP activity was not affected or minimally affected by up to 0.3 mM MgCl_2_ in the assay mixtures (Figure 5). The effects of ion chelaters EDTA and EGTA on PAP activity were measured. However, both chelators did not have significant effects on PAP activity (data not shown). This result further confirmed that the soluble PAP activity is Mg^2+^-independent in bitter melon extract. These assay results confirm the previous observations from crude extract of bitter melon and partially purified bottle gourd PAP that these PAPs are Mg^2+^-independent enzyme [39], [41]. The overall results suggest that a new class of PAP exists in bitter melon and bottle gourd which is a soluble and Mg^2+^-independent enzyme.

### Kinetic parameters of PAP

**Figure 6:**
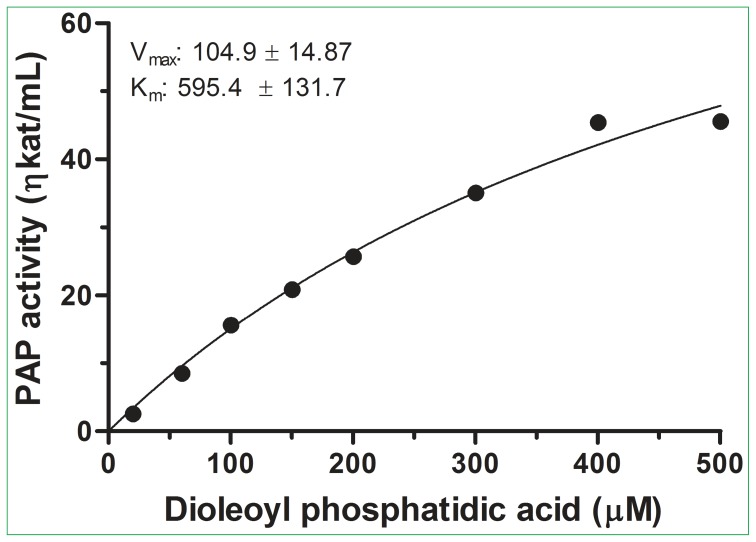
Kinetcis of PAP enzyme activity. PAP activity vs. substrate concentration. The assay was performed at 53°C, pH 6.5 for 10 min using various concentrations of DPA. The data presented are the mean of 2 assays for each sample.

The kinetic parameters of PAP were determined using the purified protein and DPA as the substrate under the optimized assay conditions (pH 6.5, 53°C and 0.3 mM MgCl_2_). The enzyme gave a typical sigmoidal curve for the substrate (Figure 6). The K*_m_* and V*_max_* values for DPA were 595.4 µM and 104.9 ηkat/mg of protein, respectively. These K*_m_* and V*_max_* values of the purified PAP from bitter melon were approximately 4-fold and 56-fold, respectively, of those obtained from crude bitter melon extract [39]. These K*_m_* and V*_max_* values of the purified PAP from bitter melon were approximately 3-fold of those obtained from partially purified bottle gourd PAP [41]. The differences in PAP kinetic parameters between the partially purified PAP and crude PAP preparations are in agreement with some observations from previous publications. It is expected that the V*_max_* values of purified or partially purified enzymes are much higher than those of the crude enzyme preparations because the V*_max_* values are calculated based on the amount of proteins used in the assays. It is also observed that the K*_m_* values of purified enzymes are higher than those of the crude enzyme preparation. For example, we observed previously that the K*_m_* value of partially purified starch synthase II is approximately 2.5-fold of those of the crude extracts [42], [43].

### Effect of phosphatase inhibitors, additives and cations on PAP activity

**Figure 7:**
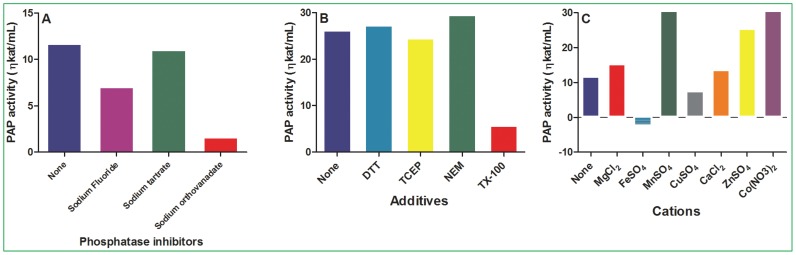
Effects of phosphatase inhibitors, additives and cations on PAP activity. The assays were performed at 53°C, pH 6.5 for 10 min using 5 µL of the PAP preparation with 500 µM DPA. (A) Effect of phosphatase inhibitors on PAP activity. The phosphatase inhibitors used were NaF (1 mM), sodium tartrate (4 mM) and sodium orthovanadate (2 mM). (B) Effect of additives on PAP activity. The final concentration of each additive in the assay buffer was 100 mM, except TX-100 was used at 10% concentration. (C) Effect of cations on PAP activity. The final concentration of each cation in the assay buffer was 0.3 mM. The data presented are the mean of 2 assays for each sample.

Three phosphatase inhibitors were used to test their effects on PAP activity. NaF partially inhibited PAP activity. Sodium orthovanadate inhibited PAP activity up to 90%. However, sodium tartrate did not affect PAP activity under the assay conditions (Figure 7A). Triton X-100 significantly reduced PAP activity but other tested additives did not affect its activity (Figure 7B). The effects of cations in the assay buffers significantly altered PAP activity. Particularly, MnSO_4_, ZnSO_4_ and Co(NO_3_)_2_ increased PAP activity, but FeSO_4_ and CuSO_4_ decreased its activity (Figure 7C).

### Native gel analysis of PAP activity

**Figure 8:**
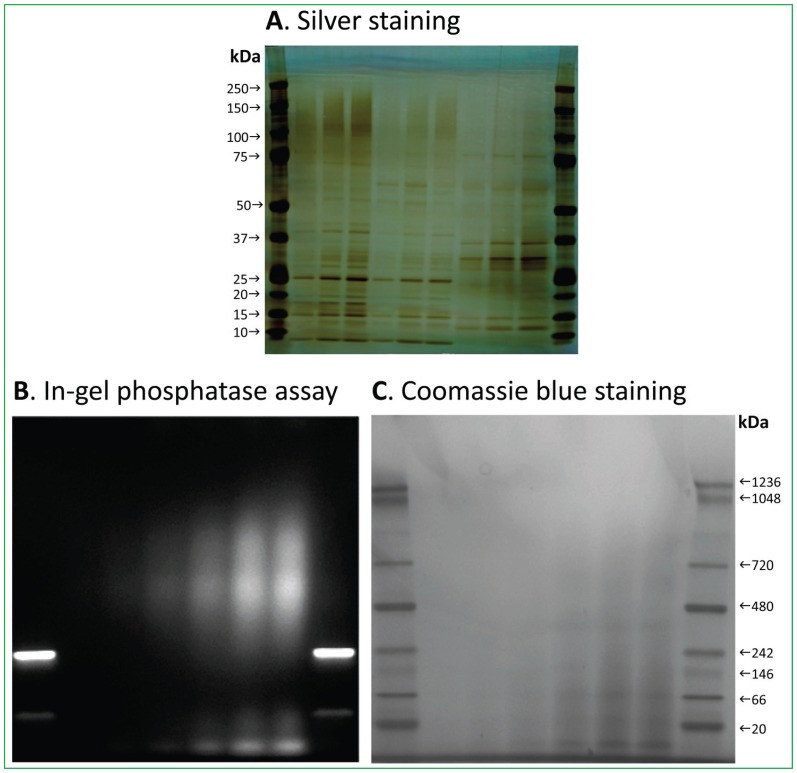
SDS-PAGE and native gel analysis of protein composition and PAP activity. (A) Denaturing gel analysis of the purified PAP fractions from Affigel Blue column by SDS-PAGE and stained by silver nitrate. Lanes 1 and 11, Bio-Rad Precision plus protein standards; lanes 2–4, 0.28, 0.55 and 0.83 µg of fraction #4; lanes 5–7, 0.19, 0.37 and 0.56 µg of fraction #5; lanes 8–10, 0.30, 0.60 and 0.90 µg of the pooled fractions 11–17. (B) PAP activity determined by in-gel phosphatase assay with DiFMUP. Lanes 1 and 8, positive control B-phycoerythrin; lanes 2–7, 0.5, 1, 3, 6, 10 and 12 µg of PAP from the pooled fraction. (C) Native gel analysis of the purified PAP fractions stained with Coomassie Blue. Lanes 1 and 8, native marker unstained protein standards; lanes 2–7, 0.5, 1, 3, 6, 10 and 12 µg of PAP from the pooled fraction.

The purified PAP fractions from Affigel Blue column contained a number of copurified proteins on SDS-PAGE (Figure 8A), but no clear protein bands were observed on the native gel (Figure 8C). PAP activity was analyzed by the in-gel phosphatase activity assay. Activity gel showed that the positive control band of B-phycoerythrin was sharp, but the activity band of PAP displayed a wide range of smear bands with much large size on the native gel (Figure 8B). These results suggest that PAP protein is probably multimerlized and/or associated with other proteins directly and/or indirectly, which is probably one of the reasons for the difficulties in further purification of PAP to homogeneity.

### Mass spectral identification of PAP-associated proteins

We attempted to identify PAP-associated proteins in the purified fractions by mass spectrometry. The PAP fractions from the second Q column and the Affigel Blue column were pooled and concentrated for tryptic and endoproteinase GluC digestions, respectively. The digested peptides were separated by LC and identified by MS/MS. MS-generated ms ions were searched against available plant sequence databases in the NCBI non-redundant protein library. LC/MS/MS identified 10 and 24 PAP-associated proteins in the trypsin and GluC digests, respectively (Table 2). MS/MS identified four of the proteins in both trypsin and GluC digests corresponding to ribosome-inactivating protein momordin I, elastase inhibitor 4, malate dehydrogenase and trypsin inhibitor 2 (Table 2). However, none of the ms corresponded to PAP sequences in other species. These MS/MS results suggest that PAP protein is probably associated with other proteins and that PAP itself is a minor component in the purified fraction.

**Table 2:** LC/MS/MS analysis of PAP-copurified proteins from bitter melon cotyledons.

No.	Co-purified proteins	Accession	Sequence	Protease
1	Ribosome-inactivating protein momordin I(Alpha-momorcharin)	P16094.2	(K)ITLPYSGNYER(L)	Trypsin
			(K)VVTSNIQLLLNTR(N)	Trypsin
			(K)YIEQQIQER(A)	Trypsin
			(K)IPIGLPALDSAISTLLHYDSTAAAGALLVLIQTTAEAAR(F)	Trypsin
			(K)VVTSNIQLLLNTR(N)	Endoproteinase Glu-C
2	Elastase inhibitor 4 (MCEI-IV)	P10296.2	(R)DSDCLAQCICVDGHCG(−)	Trypsin
			(K)RDSDCLAQCICVDGHCG(−)	Trypsin
			(R)DSDCLAQCICVDGHCG(−)	Endoproteinase Glu-C
3	Malate dehydrogenase, mitochondrial	P17783.1	(K)LALYDIAGTPGVAADVGHVNTR(S)	Trypsin
			(R)FVESSLR(A)	Trypsin
			(K)LFGVTTLDVVR(A)	Trypsin
			(D)IAGTPGVAADVGHVNTRSE(V)	Endoproteinase Glu-C
4	Trypsin inhibitor 2 (MCTI-A)	P10295.1	(R)DSDCMAQCICVDGHCG(−)	Trypsin
			(R)DSDCMAQCICVDGHCG(−)	Endoproteinase Glu-C
5	Superoxide dismutase [Cu-Zn] 1	Q42611.3	(R)AVVVHADPDDLGK(G)	Trypsin
			(R)AVVVHAEPDDLGRGGHELSKTTGNAGGR(V)	Trypsin
			(K)GGHELSLTTGNAGGR(V)	Trypsin
			(R)VACGIIGLQG(−)	Trypsin
			(K)GGHELSLTTGNAGGRVACGIIGLQG(−)	Trypsin
			(R)LACGVVGLTPV(−)	Trypsin
6	Allergen Ara h 1 (allergen Ara h I)	P43238.1	(R)IFLAGDKDNVIDQIEK(Q)	Trypsin
7	Probable lactoylglutathione, chloroplast	Q8W593.1	(R)GPTPEPLCQVMLR(V)	Trypsin
8	Peroxidase C1B	P15232.1	(R)MGNITPLTGTQGEIR(L)	Trypsin
9	Cell division protein ftsB homolog	B8GQ76.1	(K)TGLDAIEER(A)	Trypsin
10	Putative F-box/kelch-repeat protein At3g43710	Q9M2B5.1	(R)LFTLCR(R)	Trypsin
11	Enolase 2 (2-phospho-D-glycerate hydro-lyase 2)	Q9LEI9.1	(V)KVQIVGDDLLVTNPKRVE(K)	Glu-C
			(E)LRDGGSDYLGKGVSKAVE(N)	Glu-C
			(L)ELRDGGSDYLGKGVSKAVE(N)	Glu-C
12	Malate dehydrogenase, chloroplastic (pNAD-MDH)	Q9SN86.1	(D)AKAGAGSATLSMAYAAARFVE(S)	Glu-C
			(D)LPFFASR(V)	Glu-C
			(N)AFIHIISNPVNSTVPIAAE(V)	Glu-C
13	Heat shock 70 kDA protein	P26791.1	(E)TAGGVMTVLIPRNTTIPTKKE(Q)	Glu-C
14	Malate dehydrogenase, glyoxysomal	P46488.1	(E)LPFFATKVRLGRNGID(E)	Glu-C
15	Malate dehydrogenase, glyoxysomal	P19446.1	(E)LPFFASKVRLGRNGIE(E)	Glu-C
16	Fructose-biphosphate aldolase, cytoplasmic isozyme	P29356.1	(D)GGVLPGIKVDKGTVE(L)	Glu-C
17	Embryonic abundant protein 1	P46520.1	(Q)TVVPGGTGGKSLE(A)	Glu-C
18	DnaJ protein homolog (DNAJ-1)	Q04960.1	(E)ILGVSKNASQDD(L)	Glu-C
19	Nucleoside diphosphate kinase 4, chloroplastic	Q8RXA8.1	(E)IKLWFKPEE(L)	Glu-C
20	Nascent polypeptide-associated complexsubunit alpha-like protein	Q9M612.1	(D)TGVEPKDIE(L)	Glu-C
21	Glutathione reductase, chloroplastic, GR	Q43154.1	(A)QFDSTVGIHPSAAEE(F)	Glu-C
22	Ubiquitin-activating enzyme E1 (AtUBA1)	P93028.1	(V)KGGIVTQVKQPK(L)	Glu-C
23	Tetratricopeptide repeat protein 18(TPR repeat protein)	Q5T0N1.2	(Q)VVLGDSAKITVSPE(G)	Glu-C
24	Ubiquitin-activating enzyme E11	P20973.1	(E)FQDGDLVVFSE(V)	Glu-C
25	Ferredoxin -NADP reductase, rootisozyme, chloroplastic (FNR)	Q41014.2	(E)KLSQLKKNKQWHVE(V)	Glu-C
26	Peptide deformylase (PDF)	A5D1C0.1	(A)VYKIVE(L)	Glu-C
27	DNA polymerase zeta catalytic subunit (REV3-like)	Q61493.2	(K)ATSSSRSELEGRK(G)	Glu-C
28	DNA mismatch repair protein mutL	Q87VJ2.1	(V)HDFLYGTLHRALGDVRPE(N)	Glu-C
29	Trigger factor (TF)	Q55511.1	(M)AVDETKLIPVTFPE(D)	Glu-C
30	Nascent polypeptide-associated complex subunitalpha-like protein 1 (NAC-alpha-like protein 1)	Q9LHG9.1	(A)LKAADGDIVSAIME(L)	Glu-C

## Conclusions

Phosphatidic acid phosphatases (PAPs) catalyze the dephosphorylation of phosphatidic acid to diacylglycerol, the penultimate step in TAG synthesis. PAPs are widely present in plants, animals, microbes and human. PAPs are typically categorized into two subfamilies: Mg^2+^-dependent soluble PAP and Mg^2+^-independent membrane-associated PAP. In this study, we provided evidence for the existence of a new class of PAP enzyme in bitter melon (*Momordica charantia*). This class of PAP is soluble and Mg^2+^-independent. PAP protein is probably associated with other proteins in the oilseeds. Bitter melon has been used as herbal medicine for a long time. The molecular basis of these uses is supported by recent studies showing the potential of bitter melon oil being used in a wide range of nutritional and medicinal applications. Therefore, understanding and regulating PAP activities may lead to increased yield of bitter melon oil. The elucidation of PAP functions may lead to novel approaches to modulate cellular lipid storage and metabolic diseases.

## Materials and Methods

### Plant material

*Momordica charantia* (bitter melon) fruits (Indian origin) were purchased at an oriental grocery store in New Orleans, LA, USA. The fruits were collected at approximately 6 inches in length (mid-level maturity) which is at an ideal developmental stage for human consumption. The seeds were removed from the seed cavity of the fruit and washed with 0.9% ice cold saline solution. The outer coverings of the seeds were removed at room temperature using scalpel and the cotyledons removed manually.

### Preparation of tissue extract

All operations were carried out at 4°C. The seed cotyledons weighing 91 g were homogenized in 100 mL extraction buffer (50 mM NaOAc, pH 5.0, 150 mM NaCl and 10 mM MgCl_2_) using polytron tissuemizer (Tekmar Tissumizer MarkII, Cincinnati, OH) at low, medium and high speed for 30 s each. The homogenate was cooling down on ice for 1 min between bursts. The homogenate was centrifuged at 3,000 *g* for 15 min and the resulting supernatant (S1) was centrifuged at 18,000 *g* for 30 min at 4°C (Sorvall RC2B, Miami, FL). This supernatant (S2) was ultracentrifuged at 105,000 *g* for 60 min at 4°C (Sorvall Discovery 100SE, Hitachi Ltd, Tokyo, Japan) and the resulting supernatant (S3, cytosol) and the pellet (P3, microsomal membranes) were collected. The S3 supernatant was dialyzed to remove Pi from the seed extract against imidazole buffer (25 mM imidazole buffer, pH 6.5, 1 mM MgCl_2_) with three 500 mL buffer changes. The dialyzed supernatant became cloudy after dialysis, which was removed by centrifugation at 18,000 *g* for 30 min at 4°C. The final supernatant (S3-supernatant) and pellet (S3-pellet) as well as P3 were used for determining subcellular distribution of PAP activity and S3-supernatant was used for PAP purification. The protein content of the supernatant was determined by Bicinchoninic acid (BCA) method (Thermo Scientific, Rockford, IL).

### Purification of PAP

PAP purification protocol included four steps of chromatography consisting of a repeat of UNOsphere Q and S columns (Bio-Rad Laboratories, Hercules, CA). The column chromatography was performed at ambient 25°C using BioLogic LP chromatographic workstation (Bio-Rad Laboratories). During various steps of purification, active fractions were identified by PAP assay. Parallel phosphatase assay using p-nitrophenol was also carried out to confirm the purity because it was observed that PAP enzyme has phosphatase activity whereas not all phosphatases had PAP activity. All the buffers used during various stages of purification contained 1 mM MgCl_2_. Briefly, the dialyzed S3-supernatant from bitter melon cotyledon extract was applied to a 20 mL UNOsphere Q column (2.5×4.0 cm) equilibrated with the 25 mM imidazole buffer, pH 6.5 at the flow rate of 3.0 mL/min. The column was then washed with the same buffer followed by a stepwise elution with NaCl gradient in imidazole buffer (25 mM, pH 6.5) ranging from 0.2 to 0.4 M at 0.05 M NaCl increment and then with 0.5 and 1.0 M NaCl. The eluted fractions with PAP activities were combined, dialyzed against (25 mM, pH 6.5) and applied on to a 20 mL UNOsphere S column (2.5×4.0 cm) equilibrated with the same buffer at the flow rate of 3.0 mL per min. PAP activity was found in the unbound fractions. Further purification involved sequential UNOsphere Q and S columns (1 mL). The buffer and elution conditions were the same except the S column was done at pH 4.5 (25 mM acetate buffer). The PAP activity was found in the eluted fractions. The final protein with PAP activity were concentrated by ultrafiltration and used for enzyme activity assays. The concentrated proteins were further purified by Affigel Blue column (Bio-Rad Laboratories) in 25 mM imidazole, pH 6.5 and 1 mM MnSO_4_. The great majority of PAP activity was not bound to the column. The active fractions were pooled and concentrated by Amicon Ultra-0.5 mL Centrifugal Filters (Millipore Corporation, Billerica, MA) for SDS-PAGE, in-gel phosphatase activity assay and protease digestion.

### PAP activity assay

PAP activity was determined by P_i_-release assay and phosphatase assay. These activity assays for bitter melon PAP were described previously [39]. The Pi-release assay followed the ammonium molybdate-acetone-acid (AMA) method [44]. For standard assay except otherwise noted below, a 50 µL of PAP enzyme was added to 900 µL of 50 mM imidazole buffer, pH 6.5 in a 53°C water bath. The enzymatic reaction was initiated by the addition of 50 µL of PtdOH/DPA (dioleoyl-phosphatidic acid or 1,2-dioleoyl-sn-glycero-3-phosphate, sodium salt, Avanti Polar Lipids, Inc., Alabaster, Alabama), incubated for 30 min and terminated by 2 mL of AMA reagent. Citric acid (0.1 mL, 1.0 M) was added to each tube 30 s later to fix the color followed by centrifugation at 13,000 *g* for 7 min (Eppendorf 5415C, Westbury, NY). The absorbance at 355 nm was measured after blanking the spectrophotometer with the appropriate control, which was stopped at zero time. The PAP activity was expressed as nanokatals per milliliter (ηkat/mL, ηmoles orthophosphate released per sec). One International Unit (IU) is equivalent to 16.67 nkat.

To determine the pH optima of bitter melon PAP, we used 25 mM glycine-HCl (pH 1.5–3.0), 50 mM sodium acetate (pH 3.5–5.5), and 25 mM imidazole (pH 6–9). To measure the optimum temperature, the samples were incubated with substrate between 20 and 70°C in 25 mM imidazole, pH 6.5. The Mg^2+^ optima were determined by the phosphatase assay with up to 3 mM MgCl_2_ in the assay mixtures. The general procedures for PAP characterization were similar to those used for soluble starch synthases [42].

### Phosphatase activity

For phosphatase assay, PAP enzyme (2 to 10 µL) was added to a volume of 950 µL of buffer (50 mM imidazole, pH 6.5) and incubated at 53°C for 2 to 5 min with 1.25 mmole of ρ-nitrophenylphosphate (ρNPP) in a final volume of 1.0 mL. The reaction was terminated with 0.1 mL of 1.0 N NaOH and the released ρ-nitrophenol was measured spectrophotometrically at 400 nm [45].

### Kinetics of PAP

The K*_m_* and V*_max_* for bitter melon PAP using the P_i_-release assay were determined at 53°C and pH 6.5 as mentioned above. The concentration of DPA ranged from 0 to 500 µM. WindowChem’s software Enzyme Kinetics version 1.1 (Fairfield, CA) was used to compute the K*_m_* and V*_max_* values.

### Denaturing gel electrophoresis

The purified PAP enzyme preparation from Affigel Blue column was analyzed by SDS-PAGE (200 V and 70 min) using Xcell II, Mini-Cell and 4–12% Novex NuPage Bis-Tris gels with MOPS as running buffer (Life Technologies, Grand Island, NY). The separated protein bands were visualized with Pierce silver staining kit using prestained and multicolored molecular weight markers (4 to 250 kDa) as size standards (Thermo Scientific).

### Native gel electrophoresis and in-gel phosphatase activity assay

The purified PAP enzyme preparation was also analyzed by native gel for in-gel PAP activity assay using Xcell II, Mini-Cell and 3–12% Novex NuPage Bis-Tris gels with NuPAGE native running buffer (Life Technologies). The native gel was loaded with 0.5–12 µg of proteins purified from the Affigel Blue column. The protein bands on the gel were visualized with Coomassie Blue staining (Simple Blue SafeStain, Life Technologies) using native mark unstained protein standards (20 to 1236 kDa) as size standards (Thermo Scientific). PAP activity on the unstained native gel was performed using 6,8-difluoro-4-methylumbelliferyl phosphate (DiFMUP) as the substrate and B-phycoerythrin as the positive control (Life Technologies). The activity assay was performed by incubating the native gel into 3-mL reaction mixture containing 0.5 mM DiFMUP, 50 mM imidazole, pH 6.5, 3 mM MnCl_2_ and 100 mM DTT at 37°C for 5 min.

### Trypsin digestion of the purified PAP enzyme

Enzyme fraction from Affigel Blue column was concentrated in a Centricon concentrator (Amicon, centrifugal filter devices, Millipore Corporation) and during the course the imidazole buffer was exchanged to protease digestion buffer (50 mM ammonium carbonate, pH 8.0). The tryptic digests were prepared following the instructions of the in-solution tryptic digestion and guanidination kit (Thermo Scientific). Briefly, PAP-containing proteins (5–10 µg) were added to 15 µL digestion buffer containing 5.6 mM DTT in a total volume of 27 µL in a microcentrifuge tube. The digestion mixture was incubated at 95°C for 5 min. To alkylate the proteins, 3 µL of 100 mM iodoacetamide were added to the mixture and kept at room temperature in the dark. A 2 µL aliquot of activated trypsin (100 µg/µL) was added to each tube and incubated at 37°C for 3 h followed by addition of 1 µL of trypsin to the digestion mixture and incubated for another 2 h for better digestion. The digests were guanidilated to convert lysine to homoarginine by adding 10 µL of 30% NH_4_OH and 6 µL of guanidination reagent (50 mg O-methylisourea hemisulfate in 51 µL H_2_O) and incubated at 65°C for 12 min. The reaction was stopped by the addition of 3 µL of trifluoroacetic acid (TFA) and stored at –20°C before LC/MS/MS analysis.

### Endoproteinase GluC digestion of the purified PAP enzyme

Enzyme fraction from the Affigel Blue column was concentrated in a Centricon concentrator (Amicon, centrifugal filter devices, Millipore Corporation) and at the same time the buffer was exchanged to protease digestion buffer (50 mM ammonium carbonate, pH 7.8). The endoproteinase GluC digest was prepared following the manufacturer’s instructions (New England Biolabs, Boston, MA). Briefly, PAP-containing proteins (10–20 µg) were mixed with 0.5 µg endoproteinase GluC and incubated at 37°C for 16 h. The reaction was stopped by the addition of 6 µL of TFA and stored at –20°C before LC/MS/MS analysis.

### Peptide separation, mass spectral sequencing and database search

The protease digests were analyzed by LC/MS/MS consisting of an Agilent 1200 LC system, an Agilent Chip-cube interface, and an Agilent 6520 Q-TOF tandem mass spectrometer (Agilent Technologies, Santa Clara, CA). The peptides were separated using a Chip consisting of a 40 nL enrichment column and a 43 mm analytical column packed with C_18_ (5 µm beads with 300 Å pores). One-µL aliquot of the sample was transferred to the enrichment column via a capillary pump operating at a flow rate of 4 µL/min. The nano pump was operated at a flow rate of 600 nL/min. An initial gradient of 97% Solvent A (0.1% formic acid in H_2_O) and 3% Solvent B (90% acetonitrile/0.1% formic acid in H_2_O) was changed to 60% Solvent A at 12 min, 20% at 13 min, and held till 15 min. A post run time of 3 min was employed for column equilibration. The MS source was operated at 300°C with 5 L/min N_2_ flow and a fragmentor voltage of 175 V. N_2_ was used as the collision gas with collision energy varied as a function of mass and charge using a slope of 3.7 V/100 Da and an offset of 2.5 V. Both quad and Time-of-Flight (TOF) were operated in positive ion mode. Reference compounds of 322.048121 Da and 1221.990637 Da were continually leaked into the source for mass calibration. An initial MS scan was performed from m/z 300 to 1600 and up to three multiply charged ions were automatically selected for MS/MS analysis. Following the initial run, a second injection was made excluding ions previously targeted for MS/MS analysis. LC chromatograms and mass spectra were examined using Mass-Hunter software (Version B.0301; Agilent Technologies). Data files were transferred to an Agilent workstation equipped with Spectrum Mill software (Agilent Technologies). The raw MS/MS data files were extracted, sequenced and searched against the National Center for Biotechnology Information (NCBI) non-redundant protein library.
